# Prevalence of congenital hereditary sensorineural deafness in Australian Cattle Dogs and associations with coat characteristics and sex

**DOI:** 10.1186/1746-6148-8-202

**Published:** 2012-10-29

**Authors:** Susan F Sommerlad, John M Morton, Mekonnen Haile-Mariam, Isobel Johnstone, Jennifer M Seddon, Caroline A O’Leary

**Affiliations:** 1 School of Veterinary Science, The University of Queensland, Gatton, Queensland, 4343, Australia; 2 Current address: Jemora Pty Ltd, PO Box 2277, Geelong, Victoria, 3220, Australia; 3 Biosciences Research Division, Department of Primary Industries, Victorian AgriBiosciences Centre, 1 Park Drive, Bundoora, Victoria, 3083, Australia; 4 Centre for Companion Animal Health, The School of Veterinary Science, The University of Queensland, St Lucia, Queensland, 4072, Australia

## Abstract

**Background:**

Congenital hereditary sensorineural deafness (CHSD) occurs in many dog breeds, including Australian Cattle Dogs. In some breeds, CHSD is associated with a lack of cochlear melanocytes in the stria vascularis, certain coat characteristics, and potentially, abnormalities in neuroepithelial pigment production. This study investigates phenotypic markers for CHSD in 899 Australian Cattle Dogs.

**Results:**

Auditory function was tested in 899 Australian Cattle Dogs in family groups using brainstem auditory evoked response testing. Coat colour and patterns, facial and body markings, gender and parental hearing status were recorded.

Deafness prevalence among all 899 dogs was 10.8% with 7.5% unilaterally deaf, and 3.3% bilaterally deaf, and amongst pups from completely tested litters (n = 696) was 11.1%, with 7.5% unilaterally deaf, and 3.6% bilaterally deaf.

Univariable and multivariable analyses revealed a negative association between deafness and bilateral facial masks (odds ratio 0.2; *P ≤* 0.001). Using multivariable logistic animal modelling, the risk of deafness was lower in dogs with pigmented body spots (odds ratio 0.4; *P* = 0.050).

No significant associations were found between deafness and coat colour.

Within unilaterally deaf dogs with unilateral facial masks, no association was observed between the side of deafness and side of mask. The side of unilateral deafness was not significantly clustered amongst unilaterally deaf dogs from the same litter. Females were at increased risk of deafness (odds ratio from a logistic animal model 1.9; *P* = 0.034) after adjusting for any confounding by mask type and pigmented body spots.

**Conclusions:**

Australian Cattle Dogs suffer from CHSD, and this disease is more common in dogs with mask-free faces, and in those without pigmented body patches. In unilaterally deaf dogs with unilateral masks, the lack of observed association between side of deafness and side of mask suggests that if CHSD is due to defects in molecular pigment pathways, the molecular control of embryonic melanoblast migration from ectoderm to skin differs from control of migration from ectoderm to cochlea. In Australian Cattle Dogs, CHSD may be more common in females.

## Background

### Congenital hereditary sensorineural deafness

Congenital hereditary sensorineural deafness (CHSD) is a common form of deafness in dogs and has been reported in over 80 breeds, including the Dalmatian, Bull Terrier, Border Collie and Australian Cattle Dog [1]. This form of deafness can be identified in pups from about six weeks of age, and should be differentiated from sensorineural deafness due to degeneration of the auditory pathway, which usually occurs later in life, and from conductive deafness associated with aural pathology or chronic infection. Congenital hereditary sensorineural deafness can affect one or both ears, and is an all or nothing phenotype in the affected ear [1]. Bilaterally deaf pups are often identified by breeders without clinical testing, but unilateral deafness is difficult to detect without brainstem auditory evoked response (BAER) testing [2].

Congenital hereditary sensorineural deafness appears to have a variable relationship with pigmentation in different dog breeds. In breeds with solid coloured coats, such as the Doberman and Shropshire Terrier, CHSD is not associated with pigmentation and the causative lesion has been described as cochlear neuroepithelial degeneration [3,4]. In other breeds, such as the Dalmatian and Bull Terrier, CHSD is associated with a lack of coat and iris pigmentation, which is associated with absence of melanocytes in these tissues. In this latter form of deafness, there is degeneration of the stria vascularis of the cochlea during the first four weeks of life [5,6] and an absence of melanocytes in the stria of affected dogs [5,6].

### Mode of inheritance

While for many years, the mode of inheritance of CHSD has been uncertain in many breeds including the Australian Cattle Dog, results from a recent study support CHSD being inherited as an autosomal recessive trait with incomplete penetrance in the Australian Stumpy-tail Cattle Dog [7]. In that study, the deafness phenotype was associated with red-based coat colour and possibly speckling, leading to the suggestion that genes for these coat characteristics may form a tightly linked gene cluster with the gene variant associated with this form of deafness. There is evidence that this gene cluster is on *CFA10*[7]. In contrast, a variety of inheritance mechanisms have been suggested for the Dalmatian, mainly involving the interaction of several genes [8-10].

### Association of CHSD with lack of pigmentation

In several dog breeds, lack of pigmentation, rather than a specific base coat colour, has been associated with CHSD. In a study of 2,597 Border Collies, prevalence of deafness was higher in dogs with merle coat pigmentation, excessive white on the head, or blue eyes [11]. However, this contrasts with findings from a later study, where the prevalence of CHSD was not increased in Border Collies with increased white head patches [12]. In many studies in Dalmatians, the prevalence of CHSD was higher in dogs with blue eyes [1,8,9,13-16], and further, the prevalence of CHSD in Dalmatians was lower in dogs with pigmented patches in addition to pigmented spotting [1,8,13,14,16]. This may also be consistent with a recent study in the Australian Stumpy-tail Cattle Dog, a breed related to the Australian Cattle Dog, where speckled marking of the coat was weakly associated with deafness [7]. Speckled coats show an even distribution of white and red or white and black hairs [7]. To date, no study has been published investigating possible associations between CHSD and speckled or mottled coat pattern, or between CHSD and pigmented head or body patches in the Australian Cattle Dog. The only study conducted in Australian Cattle Dogs included 293 dogs, and found no significant difference in the prevalences of CHSD by coat colour (blue, red, blue and tan, or blue and black and tan) [1].

### Association with sex

Relationships between CHSD and sex have not been observed in Australian Cattle Dogs, Australian Stumpy-tail Cattle Dogs, Border Collies, Bull Terriers, and in some studies in Dalmatians [1,7,8,11]. In contrast, in other studies in Dalmatians, females were more likely to have CHSD [14,15,17]. In one study, while there was a higher prevalence of CHSD in female Dalmatians, there was also a higher prevalence of heterochromia iridis (HI) in the females compared with males [17]. Heterochromia iridis, an incomplete pigmentation of the iridial stroma, is independently associated with CHSD [17], so this finding also supports a relationship between pigment gene expression and gender. However, in another study, despite a higher prevalence of deafness in females, HI was more common in males [14]. No mechanism for this possible sex predisposition has been proposed.

### Pathogenesis and possible mechanism of CHSD in the Australian Cattle Dog

The prevalence of CHSD in breeds such as the Bull Terrier is greater in white coated dogs compared to those with coloured coats [1]. Similarly the prevalence of CHSD is higher in Dalmatians with lack of iris pigmentation giving a blue iris colouration [1]. In many other breeds, deafness prevalence is higher in dogs with a dilution of coat colour associated with the merle gene for coat colouration [1]. The lack of pigmentation occurs due to the absence of melanocytes in these areas. In Dalmatians with CHSD, there is an absence of melanocytes in the stria vascularis of the cochlea in deaf ears, and degeneration of the stria vascularis occurs in the first four weeks of the affected puppy’s life [5,6]. Melanocytes are needed for cochlear development and function, and provide specific inward and outwardly rectifying potassium channels; these are essential for strial function, production of endocochlear potential and hearing [18,19]. Thus, a defect in the genetic control of melanoblast differentiation and migration may be involved with the development of CHSD in dog breeds in which the CHSD phenotype is linked to pigmentation phenotypes.

The genetic control of melanoblast differentiation from embryonic neural crest cells, their migration and development into melanocytes in the eye, ear and skin, and their subsequent survival is complex [20]. The *S* gene [21] which controls the distribution of pigmented and white areas of the body, is strongly associated with deafness [1]. The *sw* or extreme white allele, which is present in the Dalmatian and white Bull Terrier, and the *sp* or piebald spotting allele present in other breeds, have been associated with CHSD [1]. It is probable that blue eyes in the Dalmatian, associated with an increased prevalence of CHSD, represents a strong expression of *sw*[1], whereas pigmented patches, associated with a decreased prevalence of deafness, represents a weak expression of *sw*[1]. The allele that is present in the Australian Cattle Dog is unknown. The *S* gene is now known to be the *MITF* gene [22,23], which is one of the genes that regulates melanoblast/cyte differentiation [24-26] and migration to the otic vesicles and epidermis in mice [27,28]. Thus, any association between CHSD and pigmentation may be due to changes in *MITF* and genes that code for molecules that interact in the *MITF* pathway, or in genes located near the *MITF* gene and in strong linkage disequilibrium with the CHSD phenotype [7]. In support of this, a recent study has provided some, albeit weak, support for the association of *MITF* with CHSD in the Dalmatian [29]. Hence, studies investigating coat phenotype in relation to CHSD may shed light on the pathogenesis of this disease.

### Study aims

The aims of this study were to describe the prevalence of CHSD in Australian Cattle Dogs diagnosed by using brainstem auditory response testing (BAER), and to investigate potential associations between CHSD and gender, base coat colour, speckled or mottled coat pattern, facial masks, coloured head and body spots, white patches, and parental hearing status.

## Methods

### Study overview, dog selection and brainstem auditory evoked response testing

A retrospective cross-sectional study was conducted using data from dogs tested as part of a commercial BAER-testing service. Over 6,000 dogs from 36 breeds were tested for CHSD by BAER testing at the University of Queensland over 12 years from 1996 to 2008.

The majority of dogs tested belonged to breeders and were tested as either breeding dogs or as puppies prior to sale. One hundred and fifty puppies were tested as a clinical service to private (ie non-breeder) dog owners where deafness was suspected, and none of these dogs were included in this study. Of the dogs belonging to breeders, all Australian Cattle Dog breeders that agreed to BAER test both their breeding dogs and dogs in litters that survived to six or eight weeks of age over several generations were selected for this study. All dogs from these breeders that were tested from 1996 to 2008 at the University of Queensland were enrolled in this study. These breeders exclusively used the University of Queensland for BAER testing over that period. The dogs included in the study were either from completely or incompletely tested litters of pups, usually tested when between six and eight weeks of age, or single breeding dogs from outside sources that were tested before breeding, having been untested as puppies.

Each dog was examined once. Puppies were clinically examined, then sedated with 0.03 mg/kg of acepromazine subcutaneously (Delvet Ltd, Powers Rd, Seven Hills, NSW, Australia) and 1 mg/kg pethidine HCl subcutaneously (Hamelin Pharmaceuticals, Hamelin, Germany). Healthy young dogs over six months of age were clinically examined and were sedated with 5 μg/kg medetomidine HCl intravenously (Orion Pharma Espoo, Finland). After testing this sedation was reversed with 25 μg/kg atipamezole HCl administered subcutaneously (Orion Pharma, Espoo, Finland). Older dogs underwent a clinical examination, haematology and biochemistry, and were anaesthetised with 1-2 mg /kg alfaxalone intravenously (Jurox Ltd 85 Gardiners Rd, Rutherford NSW 2073 Australia) and maintained by intubation and administration of 1-2% isoflurane (Bomac Animal Health,West Pymble, Australia).

Each BAER test was performed by one of two veterinarians trained in audiology testing (SFS and IJ) using identical protocols on the same Medelec Sapphire 2ME testing system. The electrode array used leads with 12 mm stainless steel subdermal electrodes. The array was placed subcutaneously with the active electrode at the cranial vertex on the cranial midline, and the recording electrodes placed just rostral to the base of the tragus of each ear. When one ear was being tested the electrode at the other tragus acted as the ground lead. A click signal of alternating polarity was delivered by headphones at decibel (dB) levels ranging between 30 and 80 dB nHL to an individual ear, first the left and then the right using the Medelec Sapphire 2ME testing system (Model TDH49P, Medelec Oxon UK). During testing, masking sound was administered to the non-tested ear at a level 30 dB lower than the stimulus level to prevent cross-over of signals. The filters applied gave a low frequency cut off at 100 Hz and high frequency at 5 kHz. The recorded response was the summation of 1024 clicks delivered at 11 clicks per second, and showed at least five wave-form peaks representing the auditory pathway from the inner ear and the brainstem in normal hearing dogs [2]. Ears showing a flat line waveform with no peaks visible at any decibel level were diagnosed as having CHSD [19]. Each ear was tested separately and the status of each ear recorded

### Data collection

The collection of data and data analysis for this study was approved by The University of Queensland Animal Ethics Committee, and participating breeders gave informed consent for the use of their non-identified data for this study’s research purposes.

For each dog, pedigree, date of birth, month of birth, date of BAER testing, CHSD hearing status (normal, right and left unilateral deafness, bilaterally deaf), gender, coat colour, coat markings and the presence of dark facial and body markings and white patches were recorded. The hearing statuses of sires and dams that had been tested by the authors were identified from data records. Each dog’s breeder and kennel of origin was noted, together with litter size and whether the whole litter was examined. If all puppies in the litter were not tested (ie the litter was incompletely tested), the reasons for this were noted.

Coat colour phenotypes that were recorded were base coat colour (red, blue, blue/black, blue and black and tan, blue and tan, and red and tan), coat markings (red speckling, blue speckling or mottling), the presence of dark facial and body markings and white patches. One dog was red and tan, but no dogs were chocolate or pale miscoloured. Speckling was defined in this study as an even distribution of white hairs among coloured hairs throughout the coat and mottling as a less even distribution of small patches of light hair.

**Figure 1:**
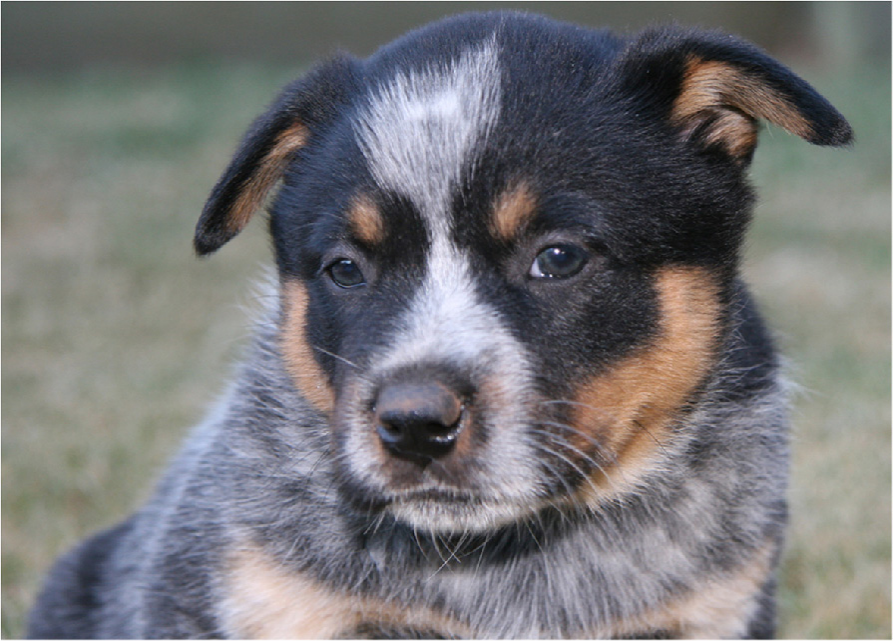
Blue Australian Cattle Dog aged 7 weeks showing a bilateral dark facial mask around both eyes.

**Figure 2:**
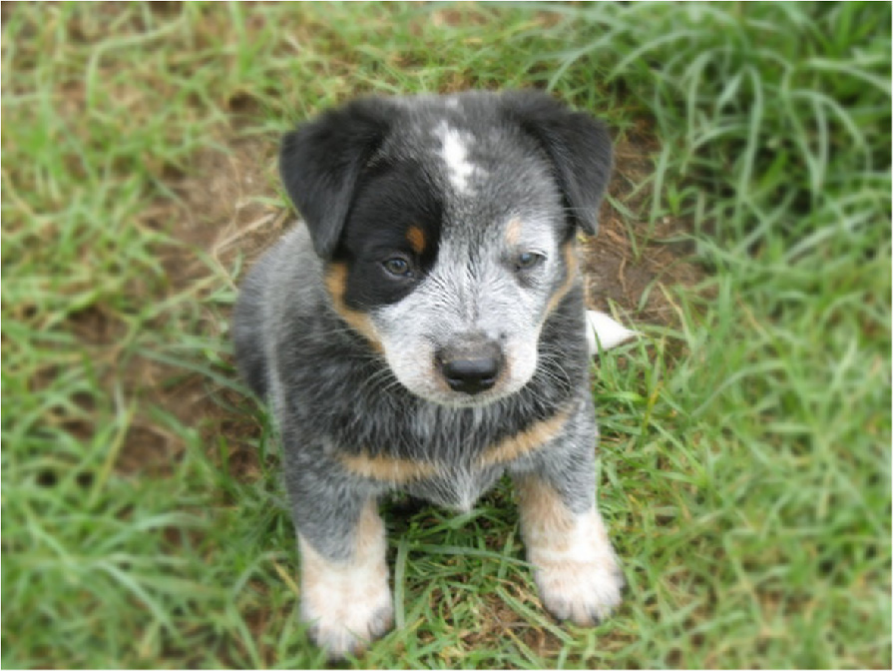
A blue Australian Cattle Dog aged 7 weeks showing a unilateral facial mask of dark hair and a white frontal “Bentley” mark on the cranium.

**Figure 3:**
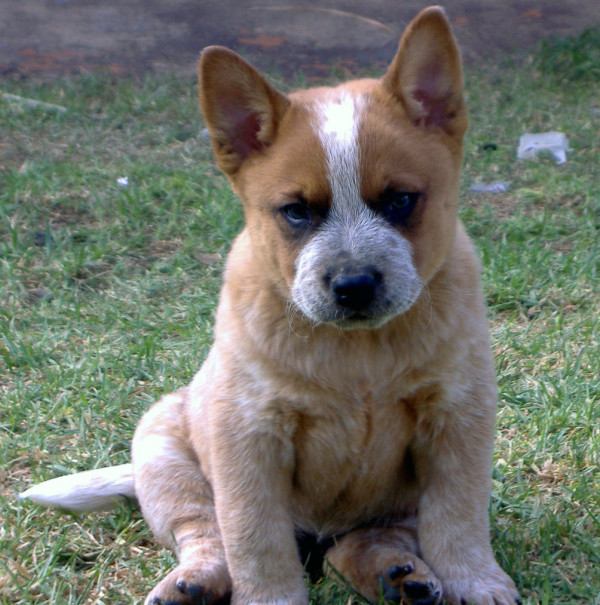
A red Australian Cattle Dog aged 6 weeks showing a bilateral facial mask of dark hair around both eyes.

**Figure 4:**
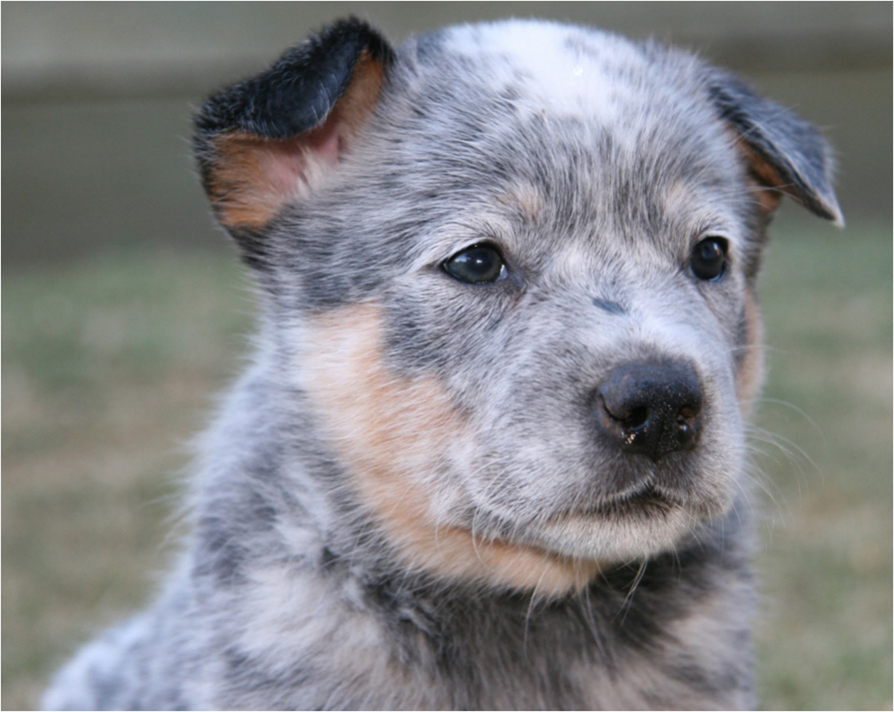
A clear- faced (no mask) blue Australian Cattle Dog aged 6 weeks.

Coloured head markings were either spots on the head, or a full mask or half mask. Masks were defined as pigmentation surrounding the eye/s and extending laterally over the temporal region (see Figures 1 and 2). Each dog was classified as having one of the following; a full bilateral mask (Figures 1 and 3), a unilateral mask (Figure 2) or a clear face with no mask (Figure 4). The coloured head markings were darker red in red dogs and dark blue/black in blue dogs. Unilateral masks were recorded as being on the left- or right-hand side.

**Figure 5:**
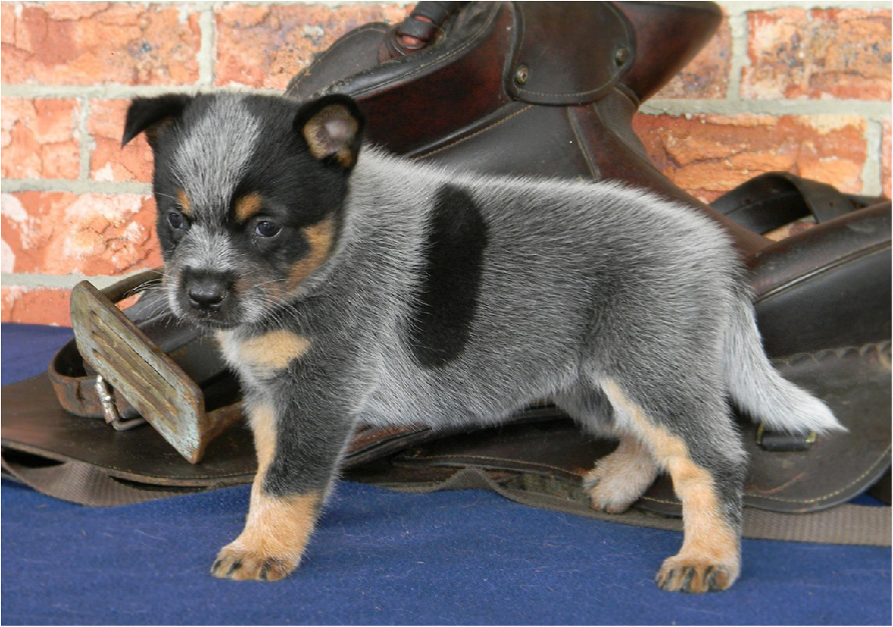
A blue Australian Cattle Dog aged 6 weeks showing a dark body spot on the flank.

Dark body patches were recorded separately (Figure 5), as were white head, body and tail patches. The white head and body patches noted were larger irregular patches and not the small white central spot or “Bentley mark” commonly seen on the frontal area of the cranium in Australian Cattle Dogs (Figure 2). All dogs had black noses and pad leather and brown/black iris colour; no blue eyes were seen.

### Statistical analyses

For all analyses, the unit of analysis was the individual dog. Amongst unilaterally deaf dogs, the proportions whose left ear was affected were described with exact 95% confidence intervals, and compared to an expected proportion of 50% under the null hypothesis using exact two-tailed *P*-values obtained from binomial goodness-of-fit tests, calculated using WinPepi (version 10.0, Abramson 2004) [30]. Two-tailed Fisher’s exact tests were used to compare proportions of dogs that were normal, unilateral and bilaterally deaf between dogs where the complete litter was tested, where part of the litter was tested, and where single breeding dogs were tested using pair-wise comparisons between these litter test categories. A two-tailed Fisher’s exact test was also used to compare proportions of subgroups of unilaterally deaf dogs with unilateral masks, namely proportions of these dogs that were deaf in the left-hand ear by side of the mask (ie left- or right-hand side). These tests were performed with WinPepi (version 10.0, Abramson 2004) [30] and disregarded correlations between dogs due to common pedigrees.

For all remaining analyses described below, we used only the 810 dogs from either completely- or incompletely-tested litters and excluded the 89 dogs tested because they were under consideration for use as breeding sires or dams. Associations between presence of deafness (at least one ear affected) and various exposure variables were assessed using logistic animal (‘threshold’) models fitted using ASReml, Release 3.0 [31]. These models used the full pedigree (ie all pedigree data that was available) and dog was fitted as a random effect with a numerator relationship matrix. Overall significance of exposure variables was assessed using Wald F-tests. Exposure variables that were associated with deafness on univariable analysis at *P* < 0.10 were fitted in a multivariable model. Mask was included even though the overall *P* value was not calculated, as the *P* value for one level was < 0.001. Heritability of this trait was estimated after fitting the multivariable model as described above.

Associations were also assessed using multilevel logistic models, fitted using Stata’s -xtmelogit- command (Stata version 11, StataCorp, College Station, Texas, USA) with three levels: dog (lowest level); litter (middle level); kennel of birth (upper level). Overall significance of exposure variables was assessed using likelihood ratio tests. Exposure variables that were associated with deafness on univariable analysis at *P* < 0.10 were fitted in a multivariable model, and the overall significance of each variable assessed, also using likelihood ratio tests. Although the univariable *P* value for sire hearing status was <0.10, this variable was not included in multivariable modelling as sire status was unknown for 298 of the 810 study dogs, and because sire hearing status was probably causally associated with other exposure variables that were fitted. Proportions of total variance associated with CHSD status (at least one deaf ear or no deaf ears) at each of the kennel- and litter-levels from the null multilevel logistic model were calculated as Var_k_/(Var_k_ + Var_l_ + π^2^/3) and (Var_k_ + Var_l_)/(Var_k_ + Var_l_ + π^2^/3), respectively, where Var_k_ and Var_l_ represent the variance at kennel- and litter-levels, respectively [32,33].

To further explore the nature of any associations between deafness and various exposure variables, univariable associations were assessed between these same factors and number of ears affected using multinomial cumulative proportion animal ‘threshold’ models fitted using ASReml, Release 3.0 [31], and univariable multinomial logistic models fitted using Stata’s -mlogit- command with standard errors adjusted for clustering by litter. With cumulative proportion models (also known as proportional odds models), where j is the category number (either 0, 1 or 2 in the current study), the exponential of the model slope coefficient estimates the odds of being in category j or higher, relative to being in a lower category. This model assumes proportional odds ie that the odds of having 1 or 2 ears affected rather than none are the same as the odds of having 2 ears affected rather than none or 1. The multinomial cumulative proportion animal models used the full pedigree (ie all pedigree data that were available) and dog was fitted as a random effect. The multinomial logistic models estimate separate relative risk ratios for having each of one or two ears affected (rather than none). Overall significance of variables was assessed using Wald tests. Only variables with at least one dog each with one and two ears affected for all exposure categories were analysed in this way.

Amongst unilaterally deaf dogs in which the side of deafness was identified, the correlation in the side affected (ie left- or right-hand side) between dogs within the same litter was explored by fitting a null multilevel logistic model using Stata’s -xtmelogit- command (Stata version 11, StataCorp, College Station, Texas, USA) with dog fitted within litter within the dog’s kennel of origin. Only unilaterally deaf dogs in which the side of deafness was identified were included in this analysis (n=60), and the outcome variable was side (ie left- or right-hand side). The proportion of total variance that was associated with CHSD status (at least one deaf ear or no deaf ears) at litter-level was then calculated as (Var_k_ + Var_l_)/(Var_k_ + Var_l_ + π^2^/3) [32,33].

## Results

### Prevalence of CHSD in various subgroups of tested dogs

In total, 899 Australian Cattle Dogs from 35 breeding kennels were enrolled in this study. These 899 study dogs consisted of 696 dogs from litters where all pups in the litter were presented for BAER testing, 114 dogs from litters where not all pups were presented for BAER testing, and 89 single dogs tested as replacement breeding stock. Common reasons for presentation of incomplete litters included the pups being dead at birth, being killed by the mother or overlain, dying due to respiratory or gastrointestinal infections, and suspected cardiac abnormality. None of the reasons for puppy mortality appeared likely to be associated with the puppies' hearing status as detected by breeders. No breeder reported suspected deafness as a reason for non-presentation of pups for testing.

Prevalences of deafness were similar for dogs tested as part of a complete litter, an incompletely-tested litter or as single dogs (Table 1). Distribution of deafness status (normal hearing, unilateral or bilateral deafness) did not differ significantly between dogs where the entire litter was tested and either where the incomplete litter was tested (*P* = 0.702) or where single dogs were tested (*P* = 0.147). However, there was some evidence that these proportions differed between dogs where incompletely-tested litters were tested compared to the testing of single dogs (*P* = 0.078), with a higher proportion of single dogs having normal hearing and lower proportions having unilateral deafness and bilateral deafness. After accounting for clustering of outcomes within litters and kennels using a multilevel logistic model, the odds of deafness (any deaf ears rather than normal hearing) were similar in dogs tested as incompletely-tested litters relative to those tested as complete litters (odds ratio 1.2; 95% CI 0.5 to 2.8; *P* = 0.693).

**Table 1:** Prevalences of congenital hereditary sensorineural deafness in Australian Cattle Dogs (and numbers of dogs affected) for dogs where the complete litter was BAER tested, for dogs from incompletely tested litters and for dogs tested individually (single dog tests)

Completeness of BAER testing of dog’s litter*	No. dogs tested**	**Normal hearing**	**Unilateral deaf**			Bilateral deaf***
			**Left-hand side**	**Right-hand side**	**Side unrecorded**	
Complete litter tested	696	88.9% (619)	3.9% (27)	3.6% (25)	0.0%	3.6% (25)
Incompletely-tested litter	114	86.8% (99)	1.8% (2)	5.3% (6)	1.8% (2)	4.4% (5)
Single dog tests	89	94.4% (84)	2.2% (2)	3.4% (3)	0.0%	0.0%
Total	899	89.2% (802)	3.4% (31)	3.8% (34)	0.2% (2)	3.3% (30)

* BAER indicates brainstem auditory evoked response test.

** No. indicates number.

*** Percentages in some rows do not sum to 100.0% due to rounding.

Of the 52 unilaterally deaf dogs where the complete litter was BAER tested, 27 were deaf in the left ear (51.9%; 95% confidence interval 37.6% to 66.0%) and this was not significantly different from 50% as expected under the null hypothesis (goodness-of-fit *P* value 0.890). Of the eight unilaterally deaf dogs from partially tested litters whose affected side was recorded, two were deaf in the left ear (25.0%; 95% confidence interval 3.2% to 65.1%) and this was not significantly different from 50% as expected under the null hypothesis (goodness-of-fit *P* value 0.289).

### Associations between potential risk factors and CHSD

Associations between exposure variables and CHSD were assessed using only the 810 dogs tested as part of completely- or incompletely-tested litters (ie we excluded single dogs tested as replacement breeding stock from these analyses). These results are shown in Tables 2 and 3 (univariable models), and Table 4 (multivariable models). Heritability of the presence of deafness (at least one deaf ear) estimated from the multivariable logistic animal model was 0.21 (standard error 0.09).

**Table 2:** Univariable associations in Australian Cattle Dogs for congenital hereditary sensorineural deafness (CHSD) from multilevel logistic models and animal models

**Variable**	No. of dogs tested*	**% normal hearing**	**% unilateral deafness**	**% bilateral deafness**^**a**^	Multilevel logistic model**		Logistic animal model***	
					**Odds ratio (95% CI)†**	***P*-value**	**Odds ratio (95% CI)†**	***P*-value**
**Sex**						**0.044**		**0.059**
Male	381	91.1%	5.8%	3.1%	Reference category		Reference category	
Female	417	86.8%	9.1%	4.1%	1.7 (1.0 to 2.8)	0.044	1.6 (1.0 to 2.6)	0.057
Not recorded	12	75.0%	16.7%	8.3%				
Colour*****						**0.785**		**0.916**
Red	327	88.7%	8.0%	3.4%	Reference category		Reference category	
Blue	137	87.6%	7.3%	5.1%	1.2 (0.6 to 2.7)	0.589	1.1 (0.5 to 2.3)	0.824
Blue black tan	319	88.7%	7.5%	3.8%	0.8 (0.4 to 1.6)	0.563	0.9 (0.5 to 1.7)	0.775
Blue tan	18	88.9%	11.1%	0.0%	0.7 (0.1 to 4.1)	0.723	0.6 (0.1 to 3.1)	0.571
Red tan	1	100.0%	0.0%	0.0%				
Blue black	6	100.0%	0.0%	0.0%				
Blue mottled	1	100.0%	0.0%	0.0%				
Red mottled	1	100.0%	0.0%	0.0%				
**Base colour**						**0.882**		**0.885**
Blue	481	88.6%	7.5%	4.0%	Reference category		Reference category	
Red	329	88.8%	7.9%	3.3%	1.0 (0.6 to 1.9)	0.882	1.0 (0.6 to 1.8)	0.890
Blue black tan						**0.447**		**0.768**
No	491	88.6%	7.7%	3.7%	Reference category		Reference category	
Yes	319	88.7%	7.5%	3.8%	0.8 (0.4 to 1.5)	0.447	0.9 (0.5 to 1.6)	0.775
Blue black								
No	804	88.6%	7.7%	3.7%	NM		NM	
Yes	6	100.0%	0.0%	0.0%				
Blue tan						**0.688**		**0.951**
No	792	88.6%	7.6%	3.8%	Reference category		Reference category	
Yes	18	88.9%	11.1%	0.0%	1.4 (0.2 to 8.5)	0.688	1.0 (0.2 to 4.7)	0.954
Red tan								
No	809	88.6%	7.7%	3.7%	NM 2		NM 2	
Yes	1	100.0%	0.0%	0.0%				
**Speckled**						**0.609**		**0.726**
No	423	88.7%	7.3%	4.0%	Reference category		Reference category	
Yes	387	88.6%	8.0%	3.4%	1.2 (0.7 to 2.1)	0.609	1.1 (0.6 to 1.9)	0.733
**Mottled**						**0.507**		**0.589**
No	805	88.7%	7.7%	3.6%	Reference category		Reference category	
Yes	5	80.0%	0.0%	20.0%	2.4 (0.2 to 33.0)	0.507	1.9 (0.2 to 20.8)	0.593
**Mask**						**0.001**		****
No	185	85.4%	12.4%	2.2%	Reference category		Reference category	
Left	66	83.3%	10.6%	6.1%	1.0 (0.4 to 2.4)	0.992	1.0 (0.4 to 2.3)	0.988
Right	58	81.0%	12.1%	6.9%	1.4 (0.6 to 3.3)	0.455	1.2 (0.5 to 2.9)	0.603
Bilateral	229	95.6%	3.1%	1.3%	0.2 (0.1 to 0.5)	0.001	0.2 (0.1 to 0.5)	<0.001
Not recorded	272	87.9%	6.6%	5.5%				
**Head spot**						**0.767**		**0.911**
No	439	88.8%	8.7%	2.5%	Reference category		Reference category	
Yes	99	89.9%	6.1%	4.0%	0.9 (0.4 to 2.0)	0.767	1.0 (0.5 to 2.0)	0.916
Not recorded	272	87.9%	6.6%	5.5%				
**Pigmented body spot**						**0.034**		**0.043**
None	430	87.4%	9.3%	3.3%	Reference category		Reference category	
At least one	104	95.2%	3.8%	1.0%	0.3 (0.1 to 0.9)	0.034	0.4 (0.1 to 1.0)	0.041
Not recorded	276	88.0%	6.5%	5.4%				
**White head/body patches**						**0.852**		**0.933**
No	508	89.0%	8.3%	2.8%	Reference category		Reference category	
Yes	27	88.9%	7.4%	3.7%	1.1 (0.3 to 4.7)	0.852	0.9 (0.3 to 3.5)	0.937
Not recorded	275	88.0%	6.5%	5.5%				
**Sire CHSD status**						**0.085**		**0.205**
Tested normal	502	91.4%	6.4%	2.2%	Reference category		Reference category	
Unilateral deaf left ear	10	70.0%	20.0%	10.0%	5.9 (0.8 to 44.0)	0.085	3.3 (0.5 to 21.1)	0.200
Unknown	298	84.6%	9.4%	6.0%				
**Dam CHSD status**						**0.321**		**0.402**
Normal	430	88.6%	8.4%	3.0%	Reference category		Reference category	
Unilateral deaf right ear	3	66.7%	0.0%	33.3%	4.8 (0.2 to 105.5)	0.321	3.4 (0.2 to 59.7)	0.400
Unknown	377	88.9%	6.9%	4.2%				

The odds ratios are for having at least one deaf ear rather than normal hearing.

*No. indicates number.

** Multilevel logistic models with dog fitted within litter within kennel of birth. Bolded *P*-values are overall likelihood ratio test *P*-values; unbolded *P*-values are Wald *P*-values for each coefficient.

*** Logistic animal (‘threshold’) models using the full pedigree (ie all pedigree data that were available), with dog fitted as a random effect with a numerator relationship matrix. Bolded *P*-values are overall Wald *P*-values; unbolded *P*-values are Wald *P*-values for each coefficient.

**** Overall p-value for mask in logistic animal model not calculated.

***** Last 5 categories were pooled for analyses.

† 95% CI indicates 95% confidence interval.

EX Dogs excluded because 0 dogs or 1 dog in group had hearing loss.

NM Variable not modelled as no dogs in group had hearing loss.

NM 2 Variable not modelled as only one dog in red tan group.

a Some rows do not sum to 100.0% due to rounding.

**Table 3:** Univariable relative risk ratios and odds ratios for each of having one or two ears affected by congenital hereditary sensorineural deafness (CHSD) rather than no ears affected in Australian Cattle Dogs from multinomial logistic models and cumulative proportion animal models

**Variable**	Multinomial logistic model*				Multinomial cumulative proportion animal model**	
	**One ear affected**		**Two ears affected**		**Odds ratio (95% CI)†**	*P*-value***
	**Relative risk ratio (95% CI)†**	*P*-value***	**Relative risk ratio (95% CI)†**	*P*-value***		
**Sex**				**0.155**		**0.069**
Male	Reference category		Reference category		Reference category	
Female	1.7 (1.0 to 2.8)	0.056	1.4 (0.6 to 3.0)	0.447	1.6 (1.0 to 2.5)	0.067
**Colour**				**0.913**		**0.940**
Red	Reference category		Reference category		Reference category	
Blue	0.9 (0.4 to 2.1)	0.857	1.5 (0.6 to 4.2)	0.402	1.1 (0.5 to 2.2)	0.878
Blue black tan	0.9 (0.5 to 1.8)	0.862	1.1 (0.4 to 3.1)	0.828	0.9 (0.5 to 1.7)	0.784
Blue tan	****				0.7 (0.1 to 3.1)*****	0.605
Red tan						
Blue black						
Blue mottled						
Red mottled						
**Base colour**				**0.886**		**0.876**
Blue	Reference category		Reference category		Reference category	
Red	1.1 (0.6 to 1.8)	0.853	0.8 (0.4 to 2.0)	0.704	1.0 (0.6 to 1.8)	0.876
Blue black tan				**0.992**		**0.783**
No	Reference category		Reference category		Reference category	
Yes	1.0 (0.5 to 1.8)	0.922	1 (0.4 to 2.5)	0.958	0.9 (0.5 to 1.6)	0.789
**Speckled**				**0.834**		**0.727**
No	Reference category		Reference category		Reference category	
Yes	1.1 (0.6 to 1.9)	0.756	0.8 (0.4 to 2.0)	0.681	1.1 (0.7 to 1.8)	0.733
**Mask**				**<0.001**		**0.002**
No	Reference category		Reference category		Reference category	
Left	0.9 (0.4 to 2.1)	0.760	2.9 (0.8 to 10.1)	0.099	0.9 (0.4 to 2.2)	0.902
Right	1.0 (0.4 to 2.6)	0.962	3.4 (0.7 to 17.0)	0.142	1.2 (0.5 to 2.7)	0.712
Bilateral	0.2 (0.1 to 0.5)	0.001	0.5 (0.1 to 2.5)	0.425	0.2 (0.1 to 0.5)	0.001
**Pigmented body spot**				**0.056**		**0.046**
None	Reference category		Reference category		Reference category	
At least one	0.4 (0.1 to 1.0)	0.052	0.3 (0.0 to 1.8)	0.176	0.4 (0.1 to 1.0)	0.044
**White head/body patches**				**0.952**		**0.899**
No	Reference category		Reference category		Reference category	
Yes	0.9 (0.2 to 4.3)	0.892	1.3 (0.2 to 11.2)	0.784	0.9 (0.2 to 3.4)	0.905
**Sire CHSD status**				**<0.001**		**0.209**
Normal tested by first-named author	Reference category		Reference category		Reference category	
Unilateral left	4.1 (1.5 to 11.1)	0.006	6.0 (1.7 to 20.9)	0.005	3.2 (0.5 to 19.1)	0.203

*Standard errors adjusted for clustering by litter.

** Multinomial cumulative proportion animal ‘threshold’ models using the full pedigree (ie all pedigree data that were available), with dog fitted as a random effect.

*** Bolded *P*-values are overall Wald *P*-values; unbolded *P*-values are Wald *P*-values for each coefficient.

**** Last 5 categories excluded from analysis as none of these dogs had both ears affected.

***** Last 5 categories were pooled for analyses.

† 95% CI indicates 95% confidence interval.

**Table 4:** Multivariable analysis showing association between congenital hereditary sensorineural deafness (CHSD) in Australian Cattle Dogs (n = 534) and sex, mask and pigmented body spots from a multilevel logistic model and an animal model

**Variable**	Multilevel logistic model*		Logistic animal model**	
	**Adjusted odds ratio (95% CI)†**	***P*-value**	**Adjusted odds ratio (95% CI)†**	***P*-value**
**Sex**		**0.032**		**0.036**
Male	Reference category		Reference category	
Female	2.0 (1.0 to 3.8)	0.035	1.9 (1.1 to 3.6)	0.034
**Mask**		**<0.001**		***
No	Reference category		Reference category	
Left	0.9 (0.4 to 2.2)	0.807	0.9 (0.4 to 2.1)	0.775
Right	1.1 (0.5 to 2.7)	0.765	1.0 (0.4 to 2.4)	0.915
Bilateral	0.2 (0.1 to 0.5)	<0.001	0.2 (0.1 to 0.5)	<0.001
**Pigmented body spot**		**0.023**		**0.052**
None	Reference category		Reference category	
At least one	0.3 (0.1 to 0.9)	0.038	0.4 (0.1 to 1.0)	0.050

Sex mask and pigmented body spot were simultaneously fitted in multivariable models. The odds ratios are for having at least one deaf ear rather than normal hearing. These analyses demonstrate that these three variables were each independently associated with CHSD.

* Multilevel logistic models with dog fitted within litter within kennel of birth. Bolded *P*-values are overall likelihood ratio test *P*-values; unbolded *P*-values are Wald *P*-values for each coefficient.

** Logistic animal (‘threshold’) models using the full pedigree (ie all pedigree data that were available), with dog fitted as a random effect with a numerator relationship matrix. Bolded *P*-values are overall Wald *P*-values; unbolded *P*-values are Wald *P*-values for each coefficient.

*** Overall p-value for mask in logistic animal model not calculated.

† 95% CI indicates 95% confidence interval.

### The prevalence of CHSD and coat markings

From both univariable and multivariable analyses, bilateral facial masks and pigmented body spots were independently associated with a reduced risk of CHSD. The prevalence of deafness in dogs with bilateral facial masks was 4.4% (3.1% unilaterally and 1.3% bilaterally deaf), compared with a prevalence of deafness of 14.6% in clear faced dogs (12.4% unilaterally and 2.2% bilaterally deaf). The odds of deafness in dogs with bilateral masks were estimated to be 0.2 times that for dogs with a clear face (*P ≤* 0.001) in both univariable and multivariable analysis. Unilateral masks were not detectably associated with a reduced risk of CHSD. The prevalence of deafness in dogs with pigmented body spots was 4.8% (3.8% unilaterally deaf and 1.0% bilaterally deaf) compared with a prevalence of 12.8% deafness (9.3% unilateral and 3.3% bilaterally deaf) in dogs without body spots. The odds of deafness in animals with pigmented body spots were estimated to be 0.3 times that for other dogs in the univariable (*P* = 0.034) and multivariable (*P* = 0.038) multilevel logistic models and 0.4 times that for other dogs in the univariable (*P* = 0.041) and multivariable (*P* = 0.05) logistic animal models. White head or body patches were not associated with an increase in the prevalence of deafness in this analysis. However, these were recorded in only 27 individuals, resulting in substantial imprecision in the estimated effect.

Fourteen dogs had unilateral masks and were unilaterally deaf on an identified side. Of these, seven dogs had masks on the left-hand side, and four of these (57.1%) were deaf on the left-hand side. The remaining seven dogs had masks on the right-hand side and three of these (42.9%) were deaf on the left-hand side. There was no significant association between the side of deafnes**s** and the side of the unilateral facial pigmented mask (*P* = 1.0).

Neither base coat colour (red, blue, blue/black, blue black and tan, blue and tan, and red and tan) nor blue or red speckled nor blue or red mottled coat patterns were significantly associated with CHSD. However, the number of mottled dogs was too small for meaningful interpretation.

### The prevalence of CHSD and sex

The prevalence of deafness in female dogs was 13.2% (9.1% unilaterally deaf and 4.1% bilaterally deaf), compared to 8.9% deafness in male dogs (5.8% unilaterally deaf and 3.1% bilaterally deaf). Female Australian Cattle Dogs had increased odds of CHSD. From the multilevel logistic models, the odds of deafness in female dogs were estimated to be 1.7 times higher than in males on univariable analysis (*P* = 0.044; Table 2), and twice as high on multivariable analysis (*P* = 0.035; Table 4). From the logistic animal models, the odds of deafness in female dogs were estimated to be 1.6 times higher than in males on univariable analysis (*P* = 0.057; Table 2), and 1.9 times as high on multivariable analysis (*P* = 0.034; Table 4). These analyses indicate that the association between sex and CHSD was probably not due to confounding by mask type or pigmented body spots.

### The prevalence of CHSD and hearing status of the sire and dam

The hearing status of the sires of study dogs was known for 63% or 512 of the 810 study dogs from completely- or partly-tested litters. The sire’s hearing status was unknown for the other dogs as the study data were collected over 12 years, and early data were incomplete regarding the deafness status of sires and dams due to the previous unavailability of BAER testing facilities in the region. The prevalence of deafness (either one or both ears affected) in offspring if the sire’s hearing was normal was 8.7%, if the sire was unilaterally deaf, was 37.5%, and if the sire’s hearing status was unknown was 16.2%. Of dogs whose sire’s hearing status was known, only 10 dogs had deaf sires so effect estimates were highly imprecise but the point estimates of the odds ratios were consistent with a strong association between hearing status of the sire and CHSD on univariable analyses (multilevel logistic model: odds ratio 5.9, *P* = 0.085; logistic animal model: odds ratio 3.3, *P* = 0.200).

The hearing status of the dams of study dogs was known for only 53% or 433 of the 810 study dogs, and only 3 dogs were known to have dams with CHSD, precluding meaningful interpretation of the association between dam’s hearing status and CHSD. However, point estimates for the odds ratios were consistent with a strong association between hearing status of the dam and CHSD.

### Analyses of number of ears affected

Odds ratios for having one ear affected (rather than none) were generally similar to those for having 2 ears affected (rather than none) (Table 3) except when considering the mask variable. For this variable, point estimates differed substantially but were quite imprecise. Odds ratios from the univariable cumulative proportion animal models (Table 3) were similar to those from the univariable logistic animal models (Table 2).

### Kennel and litter effects

Variances at kennel- and litter-levels from the null multilevel logistic model were 0.00 and 1.40 (95% CI 0.64 to 3.05), respectively. The likelihood-ratio test comparing the model to ordinary logistic regression without random effects was highly significant (P < 0.001). The proportions of total variance associated with CHSD status (at least one deaf ear or no deaf ears) at each of the kennel- and litter-levels from the null model were 0.00 and 0.30, respectively. These results indicate that there was no or little clustering of CHSD amongst dogs from different litters within the same kennel, but there was some clustering of CHSD amongst dogs from the same litter.

### Unilateral and bilateral deafness

There was no consistency in the side of unilateral deafness within a litter. In total, 60 dogs in 46 litters from 20 kennels were unilaterally deaf in an identified ear. Twenty-nine dogs were unilaterally deaf in the left ear and 31 were unilaterally deaf in the right ear. In five of the 46 litters, more than one dog was unilaterally deaf. There was no consistency in the side of unilateral deafness within a litter. Three litters each contained two unilaterally deaf dogs. In each of these litters, one pup was deaf in the left ear and one in the right. In a litter with three unilaterally deaf pups, two were deaf in the left ear and one in the right. Finally, in one litter with four unilaterally deaf dogs, three were deaf in the left ear and one in the right. The proportion of total variance associated with side of deafness at litter level from the null model was 0.14. This indicates that there was little clustering of the deaf side amongst unilaterally affected dogs from the same litter.

## Discussion

### CHSD is an inherited condition

This study provides strong evidence for the inherited nature of CHSD in the Australian Cattle Dog. Clustering of deafness amongst dogs from the same litter, but minimal clustering amongst dogs from different litters in the same kennel, is consistent with an inherited aetiology and our results were consistent with an association between hearing status of the sire and CHSD. Our estimate of the heritability of having at least one ear affected was 0.21. This result is similar to that found in Jack Russell Terriers of 0.22 [34], but lower than estimates of 0.49 in Border Collies [12] and 0.73 in Dalmations [9]. Based on these three sources of evidence, we conclude that, in the Australian Cattle Dog, as in other breeds, CHSD has a hereditary component to its aetiology, and that this condition may be controlled by a major common locus, as it appears to be in the Australian Stumpy-tail Cattle Dog where the trait appears to be likely to be autosomal recessive [7].

As with other genetic diseases, the prevalence of CHSD in the Australian Cattle Dog could be reduced by only breeding from stock with normal hearing on BAER testing and that have no deaf offspring or deaf parents. However, if the prevalence of the locus for CHSD is high, the removal of a large number of animals from the breeding programme could reduce genetic variation and lead to the exposure of other hereditary problems in the breed. The development of a molecular genetic diagnostic test could allow the gradual elimination of carriers and affected dogs from the breeding program if the frequency of the disease allele was low, thus minimising the problems associated with a small gene pool [35].

The higher prevalence of CHSD found in this study in Australian Cattle Dogs with deaf parents has also been reported in the Dalmatian [1,15,36], and in the English Setter and English Cocker Spaniel [1] Another study reported a significantly higher prevalence of deafness in offspring of untested parents (23%), compared with normal hearing parents (15%) [15], presumably because the untested population included some deaf animals. Similarly, in a study in Border Collie puppies [11], the prevalence of CHSD was significantly higher in offspring from unilaterally deaf dams (10%) compared with those from normal hearing dams (2%). In contrast, there was no significant association between parental and offspring hearing status in Australian Cattle Dogs and Bull Terriers [1], although in that study, only a few subjects had parents of known hearing status [1].

### Prevalence of CHSD

The overall prevalence of deafness in complete Australian Cattle Dog litters tested in this study was 11.1%, and the overall deafness prevalence in the 899 dogs tested was 10.8% with 7.5% unilaterally deaf and 3.3% bilaterally deaf. The prevalence of CHSD in the general Australian Cattle Dog population may be higher than this, as our study did not include individual dogs presented for suspected deafness and most of the breeders of study dogs were avoiding the use of affected animals as breeding stock. This may not be the case in the general Australian Cattle Dog population. In support of this, a higher deafness prevalence was observed in a study in the USA with 296 Australian Cattle Dogs [1] (14.5%, with 12.2% unilaterally deaf and 2.4% bilaterally deaf). The higher prevalence recorded in this clinical study could have been due to the inclusion of dogs presented for suspected deafness [1].

Unilaterally deaf dogs are difficult to identify unless they are BAER tested. Thus, the proportion of deaf dogs that are unilaterally deaf provides an estimate of the percentage of affected dogs that would probably be undetected in the absence of BAER testing [1]. The proportion for the USA study was 84% [1]. For our study, the proportion of deaf dogs that were unilaterally deaf was 0.69 (7.5/10.8), suggesting as many as 69% of affected dogs would go undetected if they were not BAER tested.

### Sex

In the current study, female dogs appeared to be at increased risk of deafness compared to males, including following adjustment of results for any confounding due to any associations between gender influence and each of mask type and pigmented body spots (Table 4). The literature is divided as to the effects of sex on the prevalence of CHSD. In some studies, no association was observed between sex and CHSD in a variety of breeds [1,8,11]. This included a study in Australian Cattle Dogs that used both binary (ie deaf/not deaf) and ordinal (normal/unilateral/bilateral) outcome variables [1]. However, in other studies, female Dalmatians were at increased risk of CHSD [14,15,17,36]. A multi-breed study found no significant sex difference in the prevalence of deafness (at least one ear affected) in Dalmatians, Bull Terriers and a small sample of Australian Cattle Dogs (n = 296), although the prevalence of CHSD was a little higher in females in all of these breeds [1]. In the same study, in English Cocker Spaniels, there was some evidence that the prevalence of CHSD differed significantly by gender (*P* = 0.035) when treated as a trichotomous trait (normal/unilateral/bilateral). When treated as a dichotomous trait (deaf/normal hearing), the p-value was higher (*P*= 0.067) [1]. In the same study in English Setters, the prevalence of deafness treated as a dichotomous trait, did not differ significantly by sex (*P* = 0.601) and distributions of the three category trait did not differ significantly by gender after accounting for data source (two subsets of English Setters were studied). Comparisons in this multi-breed study were not adjusted for potential confounders, and clustering of deafness within litter was not accounted for. The differences in the findings of these various studies may be due to variations in statistical analysis methods used, or in the relationships specific to the study populations.

### The prevalence of CHSD and coat characteristics

In our study, the presence of pigmented body spots was associated with a reduced risk of CHSD independent of gender and facial mask status. Similarly, it has been shown previously that Dalmatians with large pigmented patches instead of overall pigmented spotting are less likely to be deaf [1,8,13,14,16]. Coloured patches in Dalmatians are thought to be due to weaker expression of the *S* gene now known to be *MITF*, as all Dalmatians are homozygous for the extreme white piebald allele *sw*[1]. In Dalmatians, pigmented patches are present in the white coat at birth whereas spots are not. Similarly, Australian Cattle Dog puppies are born with white coats but also show all dark body and facial masks or markings at birth; red in the case of red dogs and blue/black in the case of blue dogs. No new markings appear as the puppy grows, and the size of the markings merely grows at the same rate as the dog. This might indicate a similar genetic basis for these markings in Dalmatians and Australian Cattle Dogs. However, other mechanisms for white markings may occur in the Australian Cattle Dog. Anecdotally Dalmatians may have been used to develop the Australian Cattle Dog as a breed but this is not well documented. Interestingly, the prevalence of CHSD in the Australian Cattle Dog could possibly be reduced if pigmented body spots were no longer classified as a show fault in this breed [37], given the association of pigmented patches with reduced deafness prevalence.

From the univariable multilevel logistic models and logistic animal models, in the current study, dogs with bilateral facial masks had a reduced risk of deafness in at least one ear. While the reduced risk of deafness in Australian Cattle Dogs with bilateral masks may be due to a weak expression of the *sw* allele of *MITF*, it is also possible that a gene other than S, possibly a locus linked to a CHSD locus, is also involved. One candidate is the *EM* allelle of the *MC1R* gene, which is associated with the presence of a facial mask in some breeds, and only a single allele is required to produce this effect [38]. It is unknown whether this gene is involved in the production of a black facial mask in the Australian Cattle Dog. However, this explanation does not account for the fact that while masks in the Australian Cattle Dog are black in blue coated dogs, masks are dark red rather than black in red coated dogs. It is also interesting that it is the bilateral rather than the unilateral mask that is associated with a reduced prevalence of deafness, possibly due to increased gene dose effects.

From results of the current study, there appeared to be no strong association between the base coat colours of red or blue, and CHSD in the Australian Cattle Dog. In a previous study on 293 Australian Cattle Dogs, prevalence of CHSD also did not differ substantially among dogs with different base coat colours. In that study, coat colours were categorised as blue, blue and black and tan, blue and tan, and red [1]. In the current study, we analysed the base coat colours in two ways, comparing deafness prevalence between (1) colour groups which had sufficiently large numbers of dogs for use in analysis; these groups were blue, blue and black and tan, blue and tan, blue and black, and red, and (2) by base colour (blue or red). Both analyses produced similar results suggesting base coat colour is not strongly associated with CHSD in the Australian Cattle Dog. Results of this and the current study are interesting as in a recent study in the related Australian Stumpy-tail Cattle Dog, a significant association was observed between coat colour and CHSD, with dogs with red coats at increased risk of CHSD compared with those having blue coats [7]. This difference in such related breeds is difficult to reconcile. While this may be due to a genuine difference between breeds, it is also possible that the study population of Australian Stumpy-tail Cattle Dogs was affected by a founder effect, resulting in a higher prevalence of CHSD in red dogs.

The number of mottled dogs (n = 5) was too small to draw any meaningful conclusions about associations between mottling and CHSD. The genetic basis for speckling and mottling is as yet unclear. The speckling effect in the Australian Cattle Dog may be associated with a variant of the *MITF* gene [22,23], or a dominant ticking gene *T*, [39]. There is also a recent description [40,41] of a flecking gene giving a roaning effect where a trait defined as roan in English Cocker Spaniels was mapped to a specific region on chromosome 38. In subsequent studies, the trait that this group defined as ‘ticking’ in English Springer Spaniels and Dalmatians also mapped to nearby regions. The authors suggested that ticking was inherited as a co-dominant trait.

This present study has identified a possible role for pigmentation genes in CHSD in the Australian Cattle Dog, due to the negative association between CHSD and masks and dark body patches. While no relationship between CHSD and white head/body patches was found, this may have been due to imprecise effect estimates due to low numbers of dogs with white body patches.

In the current study, we found no association between the side of the mask and the side of deafness. If there is truly no association, there are interesting implications for the molecular pathogenesis of CHSD in the Australian Cattle Dog. The *MITF* gene on *CFA20* regulates the differentiation of neural crest derived melanoblasts to melanocytes [24-26] and *MITF-M* and *SOX10* have been shown to be involved in melanoblast migration from the neural ectoderm to the otic vesicles and epidermis in the mouse [27,28], and are separately expressed in different cell types in the newborn cochlea [27]. Mutations of *MITF* can affect melanoblast survival and affect skin and hair pigmentation [20], and these mutations may also affect otic melanocytes and hearing status [42]. However, as it is unlikely that pigment cell migration into hair and keratinised skin are entirely controlled by the same genes [28], it may also be unlikely that melanocyte migration to the stria vascularis and to the skin and hair are totally controlled by the same genes. This explanation could account for the lack of association between the side of unilateral deafness and the side of a pigmented facial mask.

## Conclusions

Congenital hereditary sensorineural deafness is a common inherited disease in the Australian Cattle Dog and is more common in dogs with clear, mask-free faces and without pigmented body patches. Amongst unilaterally deaf dogs with unilateral dark masks, the side of the deaf ear is not strongly associated with the side of a unilateral dark mask. Thus, if CHSD is due to a defect in a molecular pigment pathway, migration of melanoblasts from the ectoderm to the skin and to the inner ear are under differing molecular control, at least during the period of the inner ear’s embryonic development in which melanoblast migration is triggered. Congenital hereditary sensorineural deafness in the Australian Cattle Dog may be more common in the female. Selection of breeding animals using BAER testing, and breeding for greater pigmentation including the presence of bilateral masks, are likely to reduce the prevalence of CHSD in this iconic Australian dog breed.

## Abbreviations

BAER: Brainstem auditory evoked response; CHSD: Congenital hereditary sensorineural deafness; *CFA* 10: Chromosome 10; dB: Decibel; kHz: Kilohertz; *MC1R*: Melanocortin 1 receptor gene; *MITF*: Microphthalmia-associated transcription factor gene; μv: Microvolt; ml: Millilitre; nHL: Normalised hearing level.

## Competing interest

None of the authors has any financial or personal relationship that could inappropriately influence or bias the content of this paper.

## Authors’ contributions

SFS contributed by the acquisition of funds, project design and creation, performed BAER testing, correlated all data, undertook drafting and revision of the manuscript. JM undertook statistical analyses, provided epidemiological advice on data management and analysis, participated in manuscript writing, and had intellectual input into manuscript revision. MH performed statistical analyses. IJ contributed by performing BAER testing, collecting of data and had intellectual input into the revision of the manuscript. JMS contributed by giving advice on data and genetic content, and was involved in the drafting and intellectual revision of the manuscript. CAO assisted in acquisition of funding, project design, advice on data creation, genetic content, drafting of the paper and intellectual input into manuscript revision and overall supervision.
